# Progesterone Exerts a Neuromodulatory Effect on Turning Behavior of Hemiparkinsonian Male Rats: Expression of 3***α***-Hydroxysteroid Oxidoreductase and Allopregnanolone as Suggestive of GABA_A_ Receptors Involvement

**DOI:** 10.1155/2015/431690

**Published:** 2015-03-31

**Authors:** Roberto Yunes, Sebastián Casas, Eliana Gaglio, Ricardo Cabrera

**Affiliations:** ^1^Instituto de Investigaciones Biomédicas (INBIOMED)-IMBECU-CONICET, Universidad de Mendoza, Huarpes 698, 5500 Mendoza, Argentina; ^2^Área de Farmacología, Facultad de Ciencias Médicas, Universidad Nacional de Cuyo, Avenida Libertador 80, Centro Universitario, 5500 Mendoza, Argentina

## Abstract

There is a growing amount of evidence for a neuroprotective role of progesterone and its neuroactive metabolite, allopregnanolone, in animal models of neurodegenerative diseases. By using a model of hemiparkinsonism in male rats, injection of the neurotoxic 6-OHDA in left striatum, we studied progesterone's effects on rotational behavior induced by amphetamine or apomorphine. Also, in order to find potential explanatory mechanisms, we studied expression and activity of nigrostriatal 3*α*-hydroxysteroid oxidoreductase, the enzyme that catalyzes progesterone to its active metabolite allopregnanolone. Coherently, we tested allopregnanolone for a possible neuromodulatory effect on rotational behavior. Also, since allopregnanolone is known as a GABA_A_ modulator, we finally examined the action of GABA_A_ antagonist bicuculline. We found that progesterone, in addition to an apparent neuroprotective effect, also increased ipsilateral expression and activity of 3*α*-hydroxysteroid oxidoreductase. It was interesting to note that ipsilateral administration of allopregnanolone reversed a clear sign of motor neurodegeneration, that is, contralateral rotational behavior. A possible GABA_A_ involvement modulated by allopregnanolone was shown by the blocking effect of bicuculline. Our results suggest that early administration of progesterone possibly activates genomic mechanisms that promote neuroprotection subchronically. This, in turn, could be partially mediated by fast, nongenomic, actions of allopregnanolone acting as an acute modulator of GABAergic transmission.

## 1. Introduction

Parkinson's disease (PD), a neurodegenerative disorder originally described in 1817 [1], affects more than 1% of the population above 55 years old, and prevalence increases to 3.1% between 75 and 84 years old [2]. The pathogeny is a primary degeneration of dopaminergic neurons in the substantia nigra (SN), although clinical features that allow medical diagnosis become evident only when neuronal death is around 80% [3]. Although precise etiology is unknown, PD has long been thought to be related to both genetic and environmental factors. Among them, oxidative stress, proteasomal dysfunction, protein aggregation, and environmental toxins, one hypothesis involves deleterious mitochondrial functionality via excitatory pathways that would lead to cytotoxicity [1, 4].

Sex steroids are molecules that, among other targets, have a great impact on the CNS. Regarding PD, it is known that men are more affected than women, at least before the menopause [5]. Strong evidence is related to the influence of estrogens (particularly 17*α*-estradiol) in preventing the disease [6], particularly if neurons are healthy at the time of the treatment [7]. Progesterone has received less attention as a potential neuroprotective molecule, even though progesterone receptors are broadly expressed throughout the whole brain, affecting cognitive capabilities and neurogenesis [8], as well as motivation and anxiolytic effects [9]. Some functions of progesterone in CNS could be attributed to its metabolite allopregnanolone (ALLO), one of the most studied neuroactive steroids [10–12]. Guennoun et al. [13] have shown that chronic administration of progesterone induces an increase in brain levels of ALLO in injured rats. This neurosteroid, a potent allosteric enhancer of GABA_A_ receptors, can increase GABAergic neurotransmission much more than benzodiazepines and barbiturates [14, 15]. ALLO is synthesized into the nervous system from progesterone through two subsequent metabolic steps. The second one is mediated by the enzyme 3*α*-hydroxysteroid oxidoreductase (3*α*-HSOR) that catalyzes reduction of dihydroprogesterone to ALLO [16–18].

We have previously demonstrated that the neuroactive steroids progesterone and ALLO modulate striatal dopaminergic activity of rats under different gonadal hormonal conditions [19, 20] and that systemic administration of progesterone to hemiparkinsonian rats prevents depression-like behavior [21]. These beneficial premotor effects led us to inquire whether or not the neuroprotective effects of progesterone and/or its metabolite ALLO on premotor conditions could be extended to motor manifestations in a model of hemiparkinsonism in rats. In this work, by using such a model of hemiparkinsonism in male rats, our objectives were to study (1) the effect of progesterone on turning behavior; (2) the potential actions of progesterone on expression and activity of nigrostriatal 3*α*-HSOR; (3) the eventual modulatory effect of intrastriatal-administered ALLO on turning behavior; and (4) the existence of some evidence regarding GABAergic transmission involvement since ALLO is a potent GABA_A_ modulatory neurosteroid.

## 2. Materials and Methods

### 2.1. Animals

Male Sprague-Dawley rats from our breeding colony (*n* = 53) were used as our subjects of study. They were 60–90 days old, 280–340 g. They were housed under controlled temperature (22 ± 2°C) and lighting (12-hour light cycle beginning at 07.30 am) conditions. Food and water were available* ad libitum*. Animals for the experiments were kept and handled according to the National Institute of Health Guide for the care and use of laboratory animals.

### 2.2. Reagents

Progesterone, ALLO, 5*α*-DHP, 6-hydroxydopamine hydrobromide (6-OHDA), amphetamine, desipramine HCl, bicuculline, apomorphine hydrochloride, and NADPH were purchased from Sigma-Aldrich (St. Louis, MO, USA). Chloral hydrate was purchased from Anedra (Buenos Aires, Argentina). The neurotoxic 6-OHDA was dissolved at a concentration of 2 *μ*g/*μ*L saline in 0.1% ascorbic acid [22]. Allopregnanolone was initially dissolved in propylene glycol to a concentration of 600 *μ*M. The concentration of ALLO used in the experiments (2 *μ*M: active in our dose response curve) was obtained by successive dilutions in sterile saline. Control animals were injected with sterile saline with 1% propylene glycol. The 4.9 *μ*M concentration of the GABA_A_ receptor antagonist bicuculline was the same used in our previous published results [20].

### 2.3. Surgical Procedures

#### 2.3.1. Left Nigrostriatal Lesion with 6-OHDA

To achieve unilateral lesions of nigrostriatal system rats were anesthetized with chloral hydrate (400 mg/kg, i.p.) and placed in a stereotaxic frame (David Kopf, USA) to inject 6-OHDA into the left striatum (Anterior-Posterior, +1.2 mm; Mediolateral, +2.5 mm; Dorsoventral, −6.5 mm relative to bregma). The neurotoxin injection was conducted to a rate of 0.5 *μ*L/min with a continuous perfusion pump. The needle was left in place for another 5 min after the injection to avoid reflux. To prevent uptake of 6-OHDA by noradrenergic neurons, animals were pretreated with desipramine (25 mg/kg, i.p.) 30–40 min before injection of 6-OHDA [22].

**Figure 1:**
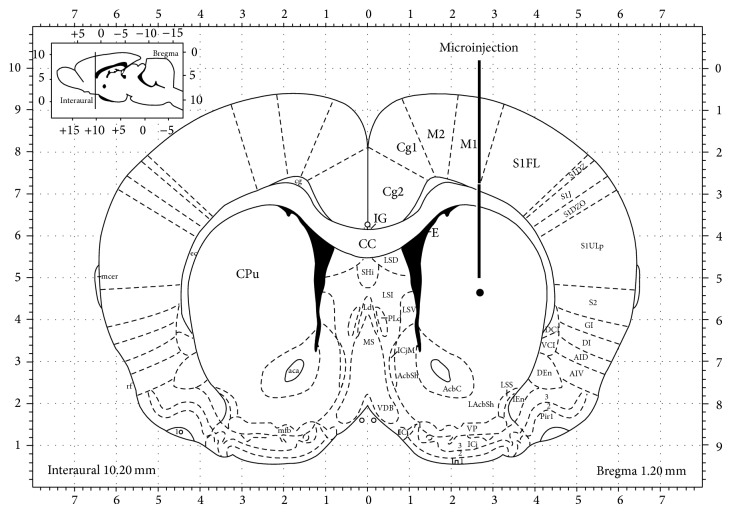
Schematic representation of a brain coronary section showing the corpus striatum and the place where microinjections were performed. Coordinates: Anterior-Posterior, +1.2 mm; Mediolateral, +2.5 mm; Dorsoventral, −6.5 mm relative to bregma. CC, corpus callosum; CPu, caudate putamen (modified from Paxinos G. and Watson C, 1997;* The Rat Brain in Stereotaxic Coordinates*, third edition).

The site of the injection was experimentally assessed by checking the striatum with a binocular microscope (Zeiss, West Germany, 4x magnification) immediately after killing animals (Figure 1). When the place of the injection was outside of the left striatum, the subjects were not further utilized.

#### 2.3.2. Striatal Cannulation

For experimental procedure II, described below, animals of different groups were anaesthetized with chloral hydrate and fixed in a stereotaxic frame. They were unilaterally implanted in left striatum with stainless steel cannula (0.80 mm × 38 mm) according to the coordinates of Paxinos and Watson's atlas (Anterior-Posterior, +1.2 mm; Mediolateral, +2.5 mm; Dorsoventral, −5.5 mm relative to bregma). The cannulae were implanted 1 mm above the structure in order to minimize damage of the brain area and fixed to the skull with dental cement. At the end of the surgery, cannulae were sealed with a stainless steel wire to protect them from obstruction. To prevent infections, all animals received an intramuscular injection of penicillin G benzathine, 1.200.000 IU each.

### 2.4. Open Field

A commercial photoelectric device (Opto-Varimex, Columbus Instruments, USA), designed to measure photobeam interruptions in individually tracked photocells, was used to assess locomotor and exploratory activity of the animals in order to avoid potentially confounding variables affecting posterior turning behavior results. The OVM consisted of a Plexiglas transparent cage (30 cm × 42 cm × 42 cm) with a homogenous black plastic floor. The walls housed infrared emitters and detectors in order to automatically register several measures: (1) horizontal activity: all movements performed on the horizontal axis; (2) ambulatory activity: all movements detected as displacement; (3) nonambulatory activity: all movements performed by the animal while remaining in the same place; (4) number of movements: number of episodic or consecutive movements performed by the animal; and (5) vertical activity: number of times the subject rises on their rear feet in the air or against the walls during at least 2 seconds. Regarding any measure other than discrete ones, for example, vertical activity, they were referred to as total counts/5 min, scoring a count as an interruption of the photobeam per second [23]. The numbers of horizontal, vertical, and total movements were recorded during a five-minute test period.

### 2.5. Experimental Design

Adult rats were randomly assigned to experimental procedure I, in order to achieve first and second objectives of this work, or experimental procedure II, to achieve third and fourth objectives (see last paragraph of Introduction).

#### 2.5.1. Experimental Procedure I

**Figure 2:**
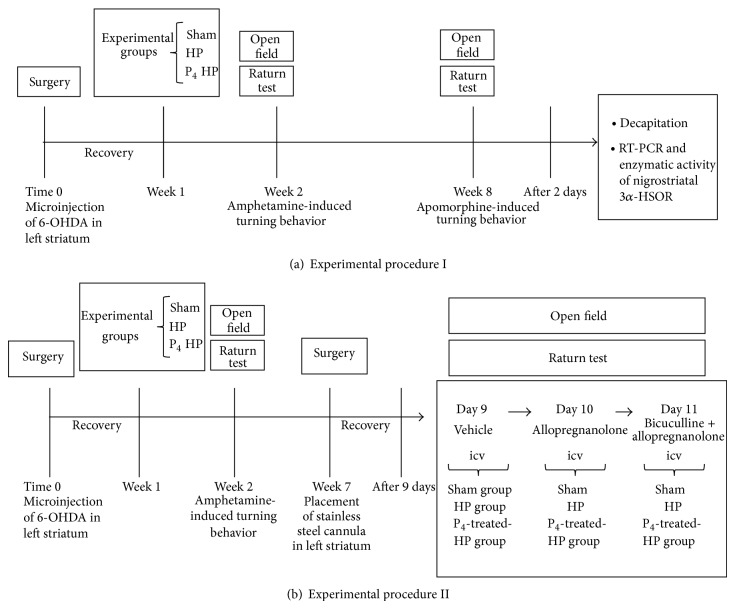
(a) Experimental procedure I: performed to evaluate the effects of progesterone on turning behavior and effects on nigrostriatal expression and enzymatic activity of 3*α*-HSOR of hemiparkinsonian rats. (b) Experimental procedure II: performed to study the effects of allopregnanolone on turning behavior of hemiparkinsonian rats. HP group (hemiparkinsonian group), P_4_-treated HP group (progesterone-treated hemiparkinsonian group). Time 0: surgery-day.

To evaluate the effect of progesterone on the turning behavior of hemiparkinsonian rats, animals were randomly assigned to one of 3 experimental groups: (1) sham group (*n* = 9) injected with vehicle, without neurotoxin; (2) hemiparkinsonian group (HP group, *n* = 11), animals were lesioned by injection of 6-OHDA in their left striatum; and (3) progesterone-treated hemiparkinsonian group (P_4_-treated HP group, *n* = 9), same as in group 2, but animals were administered at noon for three consecutive days with progesterone 4 mg/kg s.c., starting 1 week after 6-OHDA lesion [21, 24]. Two weeks later, we performed turning behavior tests after administering amphetamine, in order to ensure the use of either healthy, sham, animals or lesioned, hemiparkinsonian, animals (see Section 2.6). Eight weeks after 6-OHDA lesion, animals were tested again by administering apomorphine according to the protocol described below (see Section 2.7) [25]. Two days after the last test the animals of the three experimental groups were decapitated and their striatum and SNs dissected out for further determination of either mRNA expression by RT-PCR (sham group, *n* = 5; HP group, *n* = 5; and P_4_-treated HP group, *n* = 5) or enzymatic activity (sham group, *n* = 4; HP group, *n* = 6; and P_4_-treated HP group *n* = 4) of 3*α*-HSOR, according to the protocol described below. A schematic representation of this experimental procedure is shown in Figure 2(a).

#### 2.5.2. Experimental Procedure II

Animals were randomly assigned to one of three experimental groups: (1) sham group (*n* = 8); (2) hemiparkinsonian group (*n* = 8); and (3) progesterone-treated hemiparkinsonian group (*n* = 8). Seven weeks after 6-OHDA lesion, animals were unilaterally implanted in left corpus striatum with stainless steel cannula as described before. Nine days after cannulation, each rat was injected into left striatum for three consecutive days with the following treatments: day 9: vehicle (1 *μ*L); day 10: 2 *μ*M ALLO (1 *μ*L); and day 11: 4.9 *μ*M bicuculline + 2 *μ*M ALLO (1 *μ*L). Drug administration was performed with a needle (0.5 mm outer diameter), connected to a 10 *μ*L syringe (Hamilton, Reno, NV, USA), introduced through the guide cannula until its tip was 1.5 mm below the end of the cannula. The syringe was gently and slowly depressed for 1 min and left* in situ* for one further minute to allow diffusion from the needle throughout the striatum. Thirty minutes after drug administration, apomorphine-induced turning behavior was assayed. A schematic representation of this experimental procedure is shown in Figure 2(b).

### 2.6. Amphetamine-Induced Turning Behavior

Two weeks after receiving the neurotoxic 6-OHDA, the subjects were injected with 1 mg/kg amphetamine i.p. [22]. To run the test, the subject was placed in a plastic bowl of 20 cm of diameter. Each subject was attached to an automated rotameter (BASinc Raturn^*®*^, West Lafayette, IN) via a specially adapted harness. They were allowed to habituate to a period of dimly lit environment for 10 min before contralateral and ipsilateral turns, regarding the side of the lesion, were recorded over 60 min. The results of the turning behavior test were calculated as the difference between the numbers of contralateral and ipsilateral turns and were expressed as turns/hour [22]. Animals previously treated with 6-OHDA that failed to show ipsilateral rotations were considered as nonlesioned and therefore dismissed from the experiment.

### 2.7. Apomorphine-Induced Turning Behavior

Eight weeks after 6-OHDA lesion, apomorphine was injected s.c. at a dose of 2 mg/kg [26] and rotations were monitored for 60 min using the same experimental setup as for amphetamine-induced turning behavior. Results were expressed as turns/hour.

### 2.8. Enzymatic Activity Assay of 3-HSOR

The isolated striatum and SNs were homogenized in 2 mL of ice-cold 10 mM phosphate buffer (pH 6.5) containing 0.154 M KCI, 1 mM dithiothreitol, 0.5 mM EDTA, and 1 *μ*M PMSF. The homogenate was centrifuged at 105000 ×g for 60 min at 4°C in a Beckman Optima TL (Palo Alto, CA) ultracentrifuge equipped with a TLA-100.3 rotor. The supernatant fraction (cytosolic fraction) was stored at −80°C until the day of enzyme assay. Enzyme activity assay of 3*α*-HSOR [27] was determined spectrophotometrically by measuring the oxidation rate of NADPH at 340 nm and 37°C in a 1.0 cm-path length cuvette with a Metrolab 1600 DR (USA) spectrophotometer. The reductase activity was measured in 100 mM phosphate buffer (pH 6.5) containing 0.1 mM NADPH, 0.08 mM 5*α*-DHP (substrate), and enzyme solution in a total volume of 1.0 mL. The reaction was initiated by addition of cofactor to the assay mixture. A blank sample without substrate was routinely included. Water-insoluble substrate was dissolved in ethanol, and the final concentration of ethanol in the assay mixture did not exceed 1%, a concentration that had no effect on the catalytic activity of the enzyme. Protein concentration was determined by the method of Lowry [28] using BSA as a standard. The enzymatic activity was expressed as nmol of substrate consumed by 1 milligram of total protein in 1 minute.

### 2.9. RNA Isolation and Multiplex RT-PCR Analysis of 3*α*-HSOR

Total RNA was isolated using TRIZOL reagent (Invitrogen Life Technologies), according to the manufacturer's instructions. Gel electrophoresis and ethidium bromide staining confirmed the integrity of the samples. Quantification of RNA was based on spectrophotometric analysis at 260 nm. Two micrograms of total RNA was reverse transcribed with 200 units of MMLV Reverse Transcriptase (Promega Inc.) using hexamer random primers in a 50 *μ*L reaction mixture following manufacturer's instructions. Fragments coding for 3*α*-HOR and rat cyclophilin A (as endogenous control) were amplified by multiplex PCR with specific primers for 3*α*-HOR (5′-CAAGTGCCTTTGAATGCTGA-3′ and 5′-CCTGGAGCTCTGGTTCTTGG-3′) and rat cyclophilin A (5′-CAAGACTGAGTGGGTGGATGG-3′ and 5′-ACTTGAAGGGGAATGAGGAAA-3′), in a reaction mixture consisting of 5x Go Taq reaction buffer, 0.2 mM deoxynucleoside triphosphates, 0.6 *μ*L 3*α*-HOR (5 *μ*M) and 0.3 *μ*L (5 *μ*M) cyclophilin A primers and 0.3 *μ*L Go Taq DNA polymerase (Promega Inc.), and 7 *μ*L RT-generated cDNA in a 25 *μ*L final reaction volume [27].

### 2.10. Statistical Analysis

For the statistical analysis we utilized GraphPad Prism software for Windows (version 5.03). We performed Shapiro-Wilks' test in order to prove whether our data came from a normally distributed population. Behavioral experiments of procedure I, mRNA expression, and enzymatic activity of 3*α*-HSOR data were analyzed using a one-way ANOVA test, with a Tukey* post hoc* test. Behavioral experiments of procedure II were analyzed with a two-way ANOVA with repeated measures in a 3 × 3 factorial design, where groups (sham, HP, and P_4_-treated HP groups) and experimental conditions (vehicle, ALLO, and Bic + ALLO) were the factors. Each analysis was followed by a Bonferroni* post hoc* test. All the results were expressed as means ± SEM. The significance level to be considered as statistically different was set at *P* < 0.05.

## 3. Results

### 3.1. Effect of 6-OHDA Injection and Progesterone Treatment on the Locomotor and Exploratory Activity

Open field tests were performed to exclude eventual effect of confounding variables related to locomotor and exploratory activity of the animals. Statistical analysis showed that neither 6-OHDA, amphetamine, apomorphine, progesterone, ALLO nor Bic+ ALLO treatments modified the parameters evaluated (data not shown).

### 3.2. Experimental Procedure I

#### 3.2.1. Effect of Progesterone on the Ipsilateral Turning Behavior

**Figure 3:**
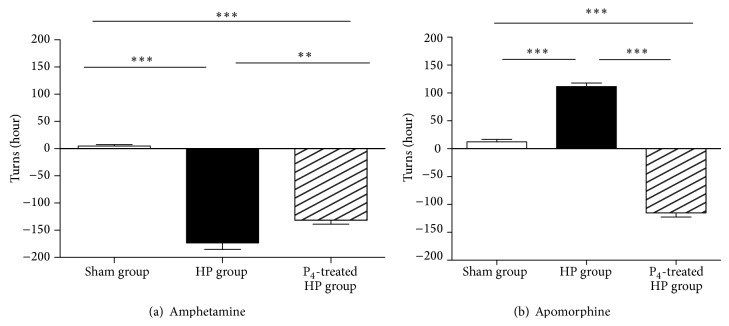
Effect of progesterone treatment on turning behavior induced by amphetamine (a) and apomorphine (b) at eight weeks after 6-OHDA lesion. Results are expressed as turns/hour (mean ± SEM); ^∗∗^ *P* < 0.01 and ^∗∗∗^ *P* < 0.001 (one-way ANOVA).

Two weeks after 6-OHDA lesion, both groups, hemiparkinsonian and progesterone-treated hemiparkinsonian groups, displayed strong ipsilateral turning behavior in response to amphetamine compared to the sham group (*P* < 0.001) (Figure 3(a)). On the other hand, the progesterone-treated hemiparkinsonian group showed a significant reduction of ipsilateral turning behavior compared to the hemiparkinsonian group (*P* < 0.01) (Figure 3(a)).

#### 3.2.2. Effect of Progesterone on the Contralateral Turning Behavior

Eight weeks following 6-OHDA lesion, hemiparkinsonian group exhibited significant contralateral turning behavior in response to apomorphine compared to sham and progesterone-treated hemiparkinsonian groups (*P* < 0.001) (Figure 3(b)). Moreover, progesterone-treated hemiparkinsonian group showed a significant ipsilateral behavior compared to sham (*P* < 0.001) and hemiparkinsonian (*P* < 0.001) groups (Figure 3(b)).

#### 3.2.3. Effect of Progesterone on 3*α*-HSOR mRNA Expression in Striatum and SN

**Figure 4:**
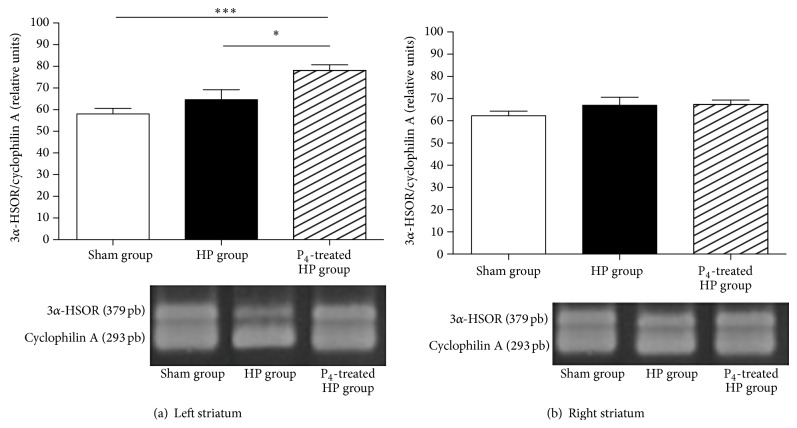
Gene expression of 3*α*-HSOR in left (a) and right striatum (b). Results are expressed as mean ± SEM of relative units cDNA of sham, HP, and P_4_-treated HP groups; ^∗^ *P* < 0.05 and ^∗∗∗^ *P* < 0.001 (one-way ANOVA). Ethidium bromide fluorescence photograph of the gel electrophoresis of the amplification products, of sham, HP, and P_4_-treated HP. The gel photographs were quantified using ImageJ software (National Institutes of Health, USA) and expressed as arbitrary units relative to cyclophilin A. HP group (hemiparkinsonian group), P_4_-treated HP group (progesterone-treated hemiparkinsonian group).

None of the experimental groups showed significant changes (data not shown) regarding 3*α*-HSOR mRNA expression of SN samples obtained 8 weeks after 6-OHDA lesion. On the other hand, the 3*α*-HSOR mRNA ipsilateral expression of progesterone-treated hemiparkinsonian group was significantly augmented compared to sham (*P* < 0.001) and hemiparkinsonian (*P* < 0.05) groups (Figure 4(a)). On the other hand, 3*α*-HSOR mRNA expression was no different among groups when SN samples contralateral to lesion were considered (Figure 4(b)).

#### 3.2.4. Effect of Progesterone on Enzymatic Activity of 3*α*-HSOR in Striatum and SN

**Figure 5:**
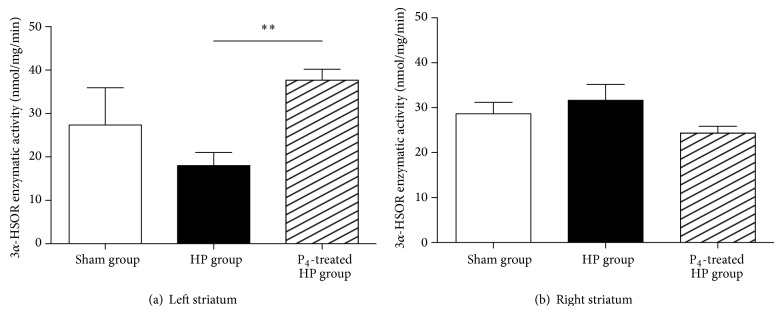
Enzymatic activity of 3*α*-HSOR in left (a) and right (b) striatum of sham, HP, and P_4_-treated HP groups. Results are expressed as a mean ± SEM of nmol/mg/min; ^∗∗^ *P* < 0.01 (one-way ANOVA). HP group (hemiparkinsonian group), P_4_-treated HP group (progesterone-treated hemiparkinsonian group).

Like 3*α*-HSOR mRNA expression, on SN samples there were no differences in the 3*α*-HSOR enzymatic activity between the different experimental groups (data not shown). On the other hand, there was a significant increase in the ipsilateral enzymatic activity of progesterone-treated hemiparkinsonian rats compared to hemiparkinsonian rats (*P* < 0.001) (Figure 5(a)). There were no statistical differences in the contralateral enzymatic activity among any of the analyzed groups (*P* > 0.05) (Figure 5(b)).

### 3.3. Experimental Procedure II

**Figure 6:**
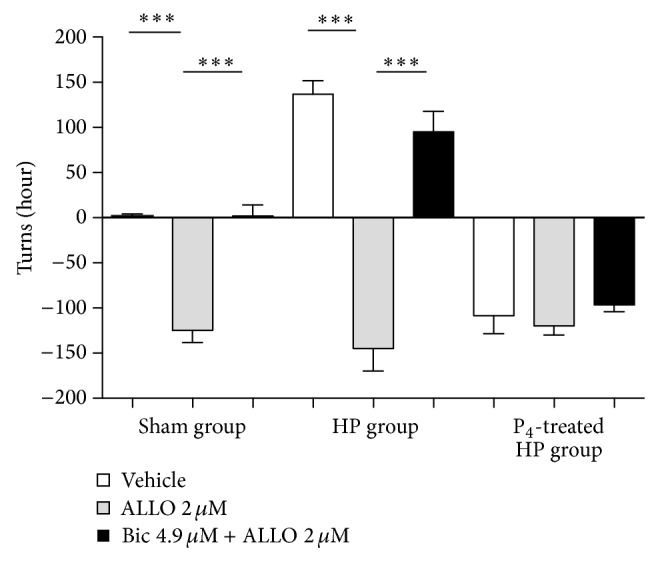
Effect of intraventricular vehicle, ALLO [2 *μ*M], and bicuculline [4.9 *μ*M] + ALLO on turning behavior induced by apomorphine at eight weeks after 6-OHDA lesion. Results are expressed as turns/hour; ^∗∗∗^ *P* < 0.001 (two-way ANOVA). ALLO: allopregnanolone, Bic: bicuculline, HP group: hemiparkinsonian group, and P_4_-treated HP group: progesterone-treated hemiparkinsonian group.

*Effect of Allopregnanolone on Turning Behavior at 8 Weeks after 6-OHDA Lesion.* Turning behavior at 8 weeks after 6-OHDA lesion was not modified by any treatment. Interestingly, the injection of ALLO induced a significant change in turning behavior to the left side in sham (*P* < 0.001) and hemiparkinsonian (*P* < 0.001) groups but not in control groups. The administration of ALLO did not show an effect on progesterone-treated hemiparkinsonian group compared with controls. The prior injection of bicuculline blocked the effects of ALLO on turning behavior in sham (*P* < 0.001) and hemiparkinsonian (*P* < 0.001) groups but interestingly did not exert any significant effect on progesterone-treated hemiparkinsonian group (*P* > 0.05) (Figure 6).

## 4. Discussion

In the present work, and in agreement with previous reports [29], hemiparkinsonian animals displayed ipsilateral turning behavior after injecting amphetamine two weeks following 6-OHDA lesion (Figure 3(a)) and contralateral turning behavior after apomorphine treatment eight weeks after lesion (Figure 3(b)). By administering progesterone one week after injection of the neurotoxic 6-OHDA, our subjects showed a reduced ipsilateral turning behavior regarding not treated ones. One possibility, although highly speculative, is that progesterone could be increasing the functionality of surviving dopaminergic neurons in the ipsilateral nigrostriatal pathway. In fact, progesterone has been reported to increase dopamine release from dopaminergic terminals [19]. Hence, amphetamine-induced dopamine release from ipsilateral nigrostriatal terminals of progesterone-treated animals could be bigger than that of those not receiving progesterone. In this sense, it could be thought as healthy remaining neurons compensating the lack of functionality induced by dysfunctional neighboring neurons. In turn, this neuromodulatory effect would be subchronically consistent since eight weeks after 6-OHDA injection turning behavior was ipsilateral. It could be possible to think of progesterone as preventing 6-OHDA-induced neuronal death through regulation of gene transcription, immunomodulation, and regulation of intracellular cascades, all of them slow mechanisms regarding interaction of the hormone with intracellular receptors [30, 31]. In brief, it looks like progesterone might prevent neuronal death. However, we cannot neglect the option of compensatory sprouting of surviving ipsilateral neurons, at the shadow of the injury, as well as compensatory upregulation. This is reinforced by the strong contralateral turning behavior shown by both injections of amphetamine or apomorphine. Of course, a combination of mechanisms could not completely be ruled out at this time.

In order to study one possible mechanism of neuroprotection, since the action of progesterone could be also mediated by its neuroactive metabolite ALLO, we were interested in the expression and enzymatic activity of nigrostriatal 3*α*-HSOR. Agís-Balboa and colleagues have reported that 3*α*-HSOR is expressed in GABAergic neurons in rat striatum [18]. Our results showed that progesterone increases both the expression and the activity of the enzyme, but only in left striatum (i.e., damaged tissue) (Figures 4 and 5). Of course, such an increase of expression and activity of the enzyme should probably be accompanied by an increase in ipsilateral striatal levels of ALLO. In fact, administration of systemic progesterone to injured rats produces increases in ALLO levels in neuronal damaged tissues, possibly reflecting a protective effect for this neuroactive steroid [32].

As it has been shown, progesterone induced expression of 3*α*-HSOR was specific of striatal tissues. Since several nongenomic actions of progesterone are accomplished by its derivative ALLO, it was interesting that this molecule produced a significant increase of ipsilateral turning behavior in all tested groups. In fact, ALLO reversed the typical contralateral turning behavior of the hemiparkinsonian animals to ipsilateral turning behavior, which is rather intriguing. Again, it is interesting to think that progesterone effect on the long term could be, at least in part, mediated by ALLO. On the other hand, all of these ALLO mediated effects were reversed by administration of bicuculline, supporting the idea of these effects being mediated by GABA_A_ receptors by allosteric modulation, although dopaminergic and glutamatergic transmission could not be conclusively excluded [11].

By taking together our results, we propose that progesterone would be playing, subchronically, an important role in dopaminergic nigrostriatal pathways in our model of hemiparkinsonism. The effect of progesterone on the expression and activity of 3*α*-HSOR suggests that ALLO could be one of the possible molecules acutely involved in the previously reported neuroprotective effect. Additionally we propose, as a working hypothesis, that ALLO, through nongenomic GABA_A_ receptor modulation, could regulate local neuroglial circuitries implicated in neuronal death. This might be an alternative cellular mechanism that could be responsible for the delay of the progressive course of the PD.

## References

[1] Dauer W., Przedborski S. (2003). Parkinson's disease: mechanisms and models. *Neuron*.

[2] Hayes M. W., Fung V. S., Kimber T. E., O'Sullivan J. D. (2010). Current concepts in the management of Parkinson disease. *Medical Journal of Australia*.

[3] Deumens R., Blokland A., Prickaerts J. (2002). Modeling Parkinson's disease in rats: an evaluation of 6-OHDA lesions of the nigrostriatal pathway. *Experimental Neurology*.

[4] Galvan A., Wichmann T. (2008). Pathophysiology of Parkinsonism. *Clinical Neurophysiology*.

[5] Shulman L. M., Bhat V. (2006). Gender disparities in Parkinson's disease. *Expert Review of Neurotherapeutics*.

[6] Bourque M., Dluzen D. E., Di Paolo T. (2009). Neuroprotective actions of sex steroids in Parkinson's disease. *Frontiers in Neuroendocrinology*.

[7] Chen S., Nilsen J., Brinton R. D. (2006). Dose and temporal pattern of estrogen exposure determines neuroprotective outcome in hippocampal neurons: therapeutic implications. *Endocrinology*.

[8] Brinton R. D., Thompson R. F., Foy M. R. (2008). Progesterone receptors: form and function in brain. *Frontiers in Neuroendocrinology*.

[9] Frye C. A. (2007). Progestins influence motivation, reward, conditioning, stress, and/or response to drugs of abuse. *Pharmacology Biochemistry and Behavior*.

[10] Wang J. M., Liu L., Irwin R. W., Chen S., Brinton R. D. (2008). Regenerative potential of allopregnanolone. *Brain Research Reviews*.

[11] Zheng P. (2009). Neuroactive steroid regulation of neurotransmitter release in the CNS: action, mechanism and possible significance. *Progress in Neurobiology*.

[12] Giuliani F. A., Yunes R., Mohn C. E., Laconi M., Rettori V., Cabrera R. (2011). Allopregnanolone induces LHRH and glutamate release through NMDA receptor modulation. *Endocrine*.

[13] Guennoun R., Labombarda F., Deniselle M. C. G., Liere P., De Nicola A. F., Schumacher M. (2015). Progesterone and allopregnanolone in the central nervous system: response to injury and implication for neuroprotection. *The Journal of Steroid Biochemistry and Molecular Biology*.

[14] Majewska M. D., Harrison N. L., Schwartz R. D., Barker J. L., Paul S. M. (1986). Steroid hormone metabolites are barbiturate-like modulators of the GABA receptor. *Science*.

[15] Morrow A. L., Suzdak P. D., Paul S. M. (1987). Steroid hormone metabolites potentiate GABA receptor-mediated chloride ion flux with nanomolar potency. *European Journal of Pharmacology*.

[16] Corpéchot C., Young J., Calvel M. (1993). Neurosteroids: 3*α*-hydroxy-5*α*-pregnan-20-one and its precursors in the brain, plasma, and steroidogenic glands of male and female rats. *Endocrinology*.

[17] Jez J. M., Penning T. M. (2001). The aldo-keto reductase (AKR) superfamily: an update. *Chemico-Biological Interactions*.

[18] Agís-Balboa R. C., Pinna G., Zhubi A. (2006). Characterization of brain neurons that express enzymes mediating neurosteroid biosynthesis. *Proceedings of the National Academy of Sciences of the United States of America*.

[19] Cabrera R. J., Bregonzio C., Laconi M., Mampel A. (2002). Allopregnanolone increase in striatal N-methyl-D-aspartic acid evoked [^3^H]dopamine release is estrogen and progesterone dependent. *Cellular and Molecular Neurobiology*.

[20] Laconi M. R., Chavez C., Cavicchia J. C. (2012). Allopregnanolone alters the luteinizing hormone, prolactin, and progesterone serum levels interfering with the regression and apoptosis in rat corpus luteum. *Hormone and Metabolic Research*.

[21] Casas S., García S., Cabrera R., Nanfaro F., Escudero C., Yunes R. (2011). Progesterone prevents depression-like behavior in a model of Parkinson's disease induced by 6-hydroxydopamine in male rats. *Pharmacology Biochemistry and Behavior*.

[22] Larramendy C., Taravini I. R. E., Saborido M. D., Ferrario J. E., Murer M. G., Gershanik O. S. (2008). Cabergoline and pramipexole fail to modify already established dyskinesias in an animal model of parkinsonism. *Behavioural Brain Research*.

[23] Nanfaro F., Cabrera R., Bazzocchini V., Laconi M., Yunes R. (2010). Pregnenolone sulfate infused in lateral septum of male rats impairs novel object recognition memory. *Pharmacological Reports*.

[24] González S. L., Labombarda F., González Deniselle M. C., Guennoun R., Schumacher M., De Nicola A. F. (2004). Progesterone up-regulates neuronal brain-derived neurotrophic factor expression in the injured spinal cord. *Neuroscience*.

[25] Frankel J. P., Lees A. J., Kempster P. A., Stern G. M. (1990). Subcutaneous apomorphine in the treatment of Parkinson's disease. *Journal of Neurology Neurosurgery and Psychiatry*.

[26] Estrella C. R., Bregonzio C., Cabrera R. J. (2002). Differential responses in central dopaminergic activity induced by apomorphine in IPL nude rat. *Behavioural Brain Research*.

[27] Escudero C., Casas S., Giuliani F. (2012). Allopregnanolone prevents memory impairment: effect on mRNA expression and enzymatic activity of hippocampal 3-*α* hydroxysteroid oxide-reductase. *Brain Research Bulletin*.

[28] Lowry O. H., Rosebrough N. J., Farr A. L., Randall R. J. (1951). Protein measurement with the Folin phenol reagent. *The Journal of Biological Chemistry*.

[29] Blandini F., Armentero M.-T., Martignoni E. (2008). The 6-hydroxydopamine model: news from the past. *Parkinsonism and Related Disorders*.

[30] Schumacher M., Guennoun R., Stein D. G., de Nicola A. F. (2007). Progesterone: therapeutic opportunities for neuroprotection and myelin repair. *Pharmacology & Therapeutics*.

[31] Guennoun R., Meffre D., Labombarda F. (2008). The membrane-associated progesterone-binding protein 25-Dx: expression, cellular localization and up-regulation after brain and spinal cord injuries. *Brain Research Reviews*.

[32] Labombarda F., Gonzalez S., Deniselle M. C. G. (2006). Progesterone increases the expression of myelin basic protein and the number of cells showing NG_2_ immunostaining in the lesioned spinal cord. *Journal of Neurotrauma*.

